# Functional characterization of unique enzymes in *Xanthomonas euvesicatoria* related to degradation of arabinofurano-oligosaccharides on hydroxyproline-rich glycoproteins

**DOI:** 10.1371/journal.pone.0201982

**Published:** 2018-08-09

**Authors:** Masayuki Nakamura, Yuino Yasukawa, Akira Furusawa, Tamao Fuchiwaki, Takashi Honda, Yuta Okamura, Kiyotaka Fujita, Hisashi Iwai

**Affiliations:** Faculty of Agriculture, Kagoshima University, Kagoshima, Japan; Nanjing Agricultural University, CHINA

## Abstract

In this study, we clarified the functions of three uncharacterized enzymes, XCV2724, XCV2728, and XCV2729, in *Xanthomonas euvesicatoria*, the causal agent of bacterial spot of tomato and pepper. The genes corresponding to the three enzymes are homologs of *hypBA1*, *hypBA2*, and *hypAA* from *Bifidobacterium longum* and are unique to *Xanthomonas* spp. among plant pathogenic bacteria. Functional characterization of the recombinant enzymes expressed using microbial systems revealed that they degrade the arabinofurano-oligosaccharides present on hydroxyproline (Hyp)-rich glycoproteins (HRGPs) such as extensin and solanaceous lectins in plant cell walls. These enzymes work coordinately to degrade the oligosaccharides. First, XeHypAA (XCV2728), belonging to the glycoside hydrolase (GH) 43 family, releases L-arabinose from L-arabinofuranose (Ara*f*)-α1,3-Ara*f*-ß1,2-Ara*f*-ß1,2-Ara*f*-ß-Hyp (Ara_4_-Hyp), cleaving its α1,3 bond; second, XeHypBA2 (XCV2729), belonging to the GH121 family, releases the disaccharide Ara*f*-ß1,2-Ara*f* from Ara*f*-ß1,2-Ara*f*-ß1,2-Ara*f*-ß-Hyp (Ara_3_-Hyp); finally, XeHypBA1 (XCV2724), belonging to GH family 127, releases L-arabinose from Ara*f*-ß-Hyp (Ara-Hyp). In summary, the main oligosaccharide structure of Ara_4_-Hyp on the HRGPs is degraded to Ara_3_-Hyp, then to Ara-Hyp, and finally to Ara monosaccharides by the action of these three enzymes. HRGPs containing oligosaccharide substrates have been reported to contribute to plant defense, and interestingly, the promoter region of the operon (*xehypBA2* and *xehypAA*) contains the plant-inducible promoter box for binding the regulator protein HrpX involved in pathogenicity. We then analyzed the expression level of the operon gene in *hrp*-inducing medium and in plants and constructed gene-deletion mutants. However, although the operon was evidently upregulated by HrpX, three single-gene deletion mutants (Δ*xehypBA1*, Δ*xehypBA2*, Δ*xehypAA*) and even a triple-gene deletion mutant (Δ*xehypBA1-BA2-AA*) remained pathogenic, and had no effect on nonhost resistance, either, indicating that these three enzymes are not involved in either pathogenicity or nonhost resistance reactions. This is the first report of enzymes in plant pathogenic bacteria that catalyze the degradation of Hyp-linked-L-arabinofuranosides in plant cell walls.

## Introduction

**Fig 1:**
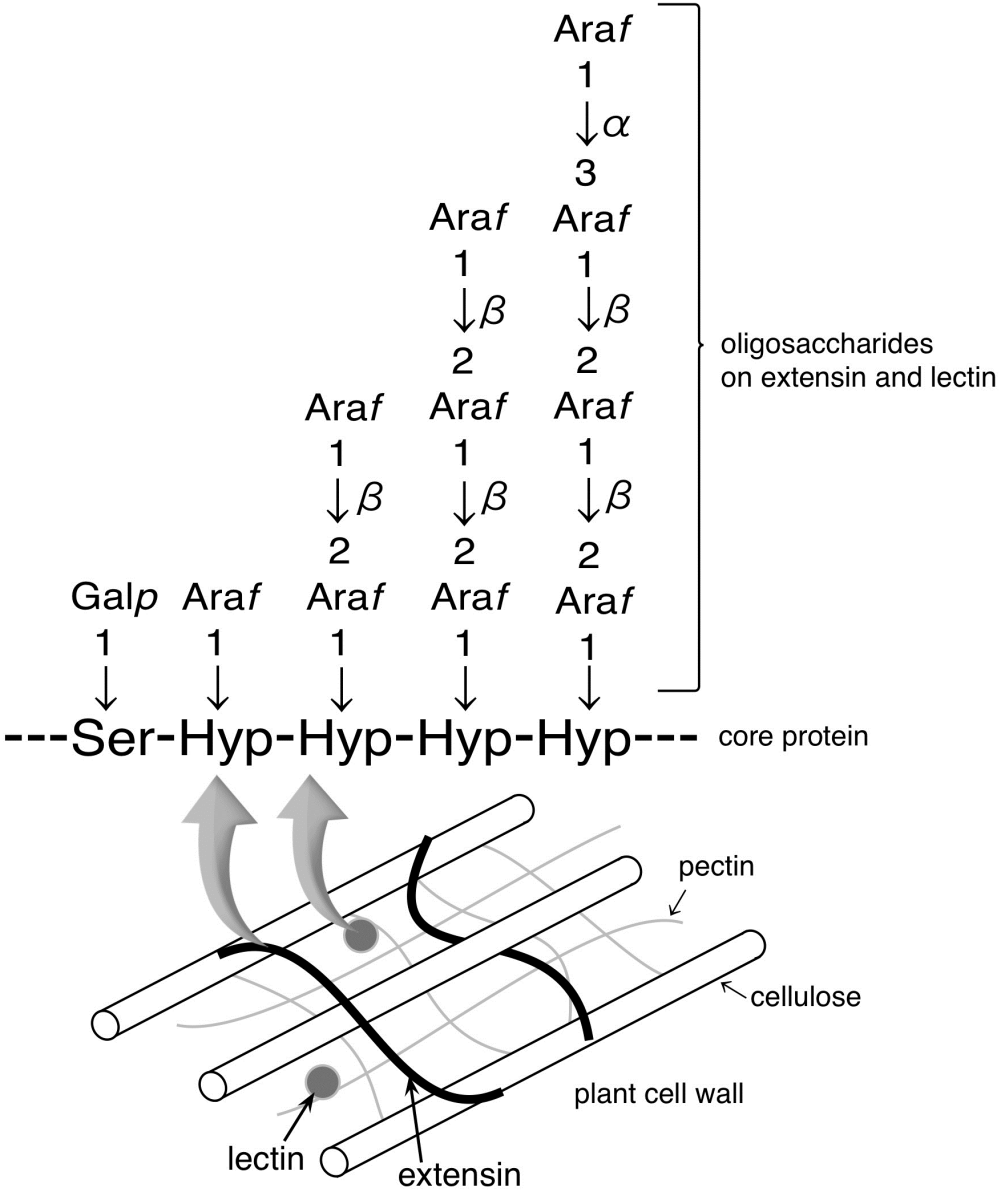
Structure of arabino-oligosaccharides on extensin and solanaceous lecins in plant cell walls. Hydoroxyproline (Hyp) residues are *O*-glycosylated with 1–4 arabinofuranosyl (Ara*f*) residues with ß-l-arabinofuranosyl linkages. These proteins contain repetitive Ser-Hyp_4_ motifs.

Three novel glycoside hydrolases GH 127 ß-l-arabinofuranosidase (HypBA1) [1], GH 121 ß-l-arabinobiosidase (HypBA2) [2], and GH43 α-l-arabinofuranosidase (HypAA) [unpublished data] in the gut bacterium *Bifidobacterium longum* were recently reported. The three enzymes degrade arabinofurano-oligosaccharides on hydroxyproline-rich glycoproteins (HRGPs) such as extensin and solanaceous lectins, thus providing the bacterium with L-arabinose as a carbon source from HRGPs that reach the intestine [1, 2]. HRGPs are found in plant cell walls, and their synthesis can be induced during plant defense against a pathogen [3–5]. Inter- and intramolecular cross-linking of extensin forms a highly linked network as a major structural component of plant cell walls and a barrier to pathogen ingress [6–9]. Lectins, which recognize and bind specific carbohydrates, can also function in plant defense signaling and responses to pathogens [10–13]. Extensin and solanaceous lectins contain repetitive serine (Ser)-hydroxyproline (Hyp)_4_ motifs with Hyp residues that are *O*-glycosylated with 1–4 arabinofuranosyl (Ara*f*) residues with ß-l-Ara*f* linkages (Fig 1). The structures of Ara_3_-Hyp and Ara_4_-Hyp, which are the major constituents of Hyp-linked ß-l-arabinofuranosides in dicotyledons [14–16], are Ara*f*-ß1,2-Ara*f*-ß1,2-Ara*f*-ß-Hyp and Ara*f*-α1,3-Ara*f*-ß1,2-Ara*f*-ß1,2-Ara*f*-ß-Hyp, respectively. HypAA from *B*. *longum* releases L-arabinose from Ara_4_-Hyp by cleaving the α1,3 bond [unpublished data]. HypBA2 liberates Ara*f*-ß1,2-Ara*f* (ß-Ara_2_) from Ara_3_-Hyp [2]. HypBA1 releases L-arabinose from ß-Ara_2_, Ara*f*-ß-Hyp (Ara-Hyp), Ara*f*-ß1,2-Ara*f*-ß-Hyp (Ara_2_-Hyp), and Ara_3_-Hyp [1].

Fujita et al. [1, 2] searched the Pfam database for homologs of the three novel genes encoding HypBA1, HypBA2 and HypAA from *B*. *longum*, and interestingly, homologs were found only in the genomes of *Xanthomonas* spp. among plant pathogenic bacteria. Our interest was piqued because the enzymes from *B*. *longum* work on sugar chains of HRGPs that contribute to plant defense [1, 2]. In addition, *xcv2729* from *X*. *euvesicatoria*, corresponding to the homolog of *hypBA2*, is expressed inductively by HrpG and HrpX [17], two upstream regulators of *hrp* genes encoding the type III secretion system (a membrane-embedded nanomachine) that is essential for pathogenicity in *Xanthomonas* spp. [18, 19]. Therefore, to investigate whether these enzymes from *Xanthomonas* spp. have the same functions as those of *B*. *longum* and are involved in pathogenicity in this study, we cloned the homologous genes and characterized the recombinant enzymes from *X*. *euvesicatoria* (formerly *X*. *campestris* pv. *vesicatoria*), which causes bacterial leaf spot [20, 21]. This bacterium infects solanaceous plants, such as tomato and pepper, that contain both extensin and solanaceous lectins. This is the first report of enzymes that catalyze the degradation of Hyp-linked-L-arabinofuranosides in plant pathogenic bacteria. We also discuss why *X*. *euvesicatoria* may have these three unique enzymes.

## Materials and methods

### Bacterial strains, growth conditions and plasmids

The bacterial strains and plasmids used in this study are listed in Table 1. *X*. *euvesicatoria* UPB139 corresponds to strain 85–10 in the KEGG database (T00288) [20–22] and was grown at 28°C using complex nutrient-yeast-glycerol medium (NYG) [23] or *hrp*-inducing medium (XVM2) that provides an environment similar to the plant extracellular space [24]. *Escherichia coli* strains were grown at 37°C in Luria-Bertani broth (LB) [25] for all routine purposes. *Brevibacillus choshinensis* was grown at 30°C using TM medium [26]. Antibiotics were added to media at the following final concentrations: 100 μg/mL ampicillin, 25 μg/mL kanamycin, and 50 μg/mL neomycin.

**Table 1:** Bacterial strains and plasmids used in study.

Strains/plasmids	Relevant characteristics	Reference/source
*Xanthomonas euvesicatoria*		
UPB139	Wild type isolated from tomato	[22]
Δ*hrpX*	*hrpX* deletion mutant of UPB139	This study
Δ*xehypBA1*	*xehypBA1* deletion mutant of UPB139	This study
Δ*xehypBA2*	*xehypBA2* deletion mutant of UPB139	This study
Δ*xehypAA*	*xehypAA* deletion mutant of UPB139	This study
Δ*xehypBA2-AA*	*xehypBA2* and *xehypAA* deletion mutant of UPB139	This study
Δ*xehypBA1-BA2-AA*	*xehypBA1*, *xehypBA2* and *xehypAA* deletion mutant of UPB139	This study
*Escherichia coli*		
XL1-Blue	*hsdR*17, *supE*44, *recA*1, *endA*1, *gyrA*46, *thi*, *relA*1, *lac*/F’ {*proAB*^+^, *lac I*^q^, *lacZ*ΔM15:: Tn*10*(*tet*^r^)}	Agilent
JM109	*ecA*1, *endA*1, *gyrA*96, *thi*, *hsdR*17(r_K_^-^ m_K_^+^),*e*14^-^ (*mcrA*^-^), *supE*44, *relA*1, Δ (*lac-proAB*)/F’{*traD*36, *proAB*^+^, *lacI*^q^, *lacZ*ΔM15}	Takara Bio
BL21 (λDE3)	F^-^, *ompT*, *hsdS*_B_(r_B_^-^ m_B_^-^), *gal*(*λc*I 857, *ind*1, *Sam*7, *nin*5, *lacUV*5-T7*gene*1), *dcm*(DE3)	Merck
S17-1 (*λpir*)	*hsdR*, *recA*, *pro*, RP4-2 (Tc::Mu; *Km*::Tn7) (*λ pir*)	Biomedal
*Brevibacillus choshinensis*		
SP3	*imp*, *em-*, *spoIIAC*	Takara Bio
Plasmids		
pET-23b (+)	Expression vector, Ap^r^, His-tag	Merck
pBIC3	Expression vector, P22 promoter, P22 signal peptide, Nm^r^, His-tag	Takara Bio
pCold TF	Expression vector, trigger factor, Ap^r^, His-tag	Takara Bio
pK18mobSacB	Mobilizable cloning vector; pUC-oriV, *mob*, *sacB*, Km^r^	[27]
pET23-xcv2724	Expression vector for XeHypBA1 in pET-23b (+)	This study
pBIC3-xcv2728	Expression vector for XeHypAA in pBIC3, Nm^r^	This study
pCold-xcv2729	Expression vector for XeHypBA2 in pCold TF, Ap^r^	This study
pK18-hrpX	Deletion vector for *hrpX* in pK18mobSacB, Km^r^	This study
pK18-xcv2724	Deletion vector for *xehypBA1* in pK18mobSacB, Km^r^	This study
pK18-xcv2728	Deletion vector for *xehypAA* in pK18mobSacB, Km^r^	This study
pK18-xcv2729	Deletion vector for *xehypBA2* in pK18mobSacB, Km^r^	This study
pK18-xcv2728-2729	Deletion vector for *xehypAA* and *xehypAA* in pK18mobSacB, Km^r^	This study
pK18-xcv2724-2728-2729	Deletion vector for *xehypBA1*, *xehypAA* and *xehypBA2* in pK18mobSacB, Km^r^	This study

Ap^r^, ampicillin resistance; Nm^r^, neomycin resistance; Km^r^, kanamycin resistance

### Construction of protein expression vectors

The genomic DNA of *X*. *euvesicatoria* UPB139 was extracted using a NucleoSpin Microbial DNA kit (Takara Bio, Otsu, Japan) and used for further PCR amplification. The primers used in this work are all shown in S1 Table. A fragment of *xcv2724*, encoding amino acids (aa) 44 to 791, was amplified with primer set PEX1/PEX2 to eliminate the N-terminal signal peptide (N-sp). The amplified fragment was cloned into pET-23b digested with *Nde*I and *Xho*I using an In-Fusion HD Cloning kit (Takara Bio), yielding pET23-xcv2724. A fragment of *xcv2728*, encoding aa 25 to 528, was amplified with primer set PEX5/PEX6 without N-sp. The amplified fragment was cloned into the linearized pBIC3 using the *Brevibacillus in vivo* cloning (BIC) method [28, 29]; briefly, a mixture of the PCR product and the linearized plasmid is directly transferred into *B*. *choshinensis* competent cells in which the insert and the plasmid are spontaneously combined via homologous recombination. Thus, the expression vector of *xcv2728* was constructed without using *E*. *coli*, resulting in pBIC3-xcv2728. A fragment of *xcv2729*, encoding aa 43 to 1452, was amplified with primer set EXP3/EXP4 without N-sp and cloned into pCold TF DNA digested with *Nde*I and *Xho*I using an In-Fusion HD Cloning kit (Takara Bio), yielding pCold-xcv2729. The cloned inserts were sequenced with an ABI 3100 DNA sequencer using a BigDye Terminator 3.1 Cycle Sequencing Kit (Applied Biosystems, Foster City, CA).

### Expression and purification of recombinant protein

For the expression of XeHypBA1, vector pET23-xcv2724 was transferred into *E*. *coli* BL21 (λDE3) cells, then a single colony was added to 50 mL LB containing 50 mg/mL kanamycin and grown to an OD_600_ of 0.6 at 37°C. The culture was then induced with 1 mM isopropyl ß-d-1-thiogalactopyranoside (IPTG) for 18 h at 15°C. For the expression of XeHypBA2, vector pCold-xcv2729 was transferred into *E*. *coli* BL21 (λDE3) cells, and the protein fused with the trigger factor chaperone was expressed as described above. For the expression of XeHypAA, vector pBIC3-xcv2728 was transferred into *B*. *choshinensis* SP3, and a single colony was grown in 50 mL TM containing 50 mg/mL neomycin at 30°C for 48 h.

Expressed proteins were purified with the MagneHis Protein Purification System (Promega, Madison, WI) or the Capturem His-Tagged Purification kit (Takara Bio) and desalted with the Zeba Spin Desalting Columns 7K MWCO (Thermo Scientific, Rockford, IL, USA). The purified proteins were confirmed by sodium dodecyl sulfate polyacrylamide gel electrophoresis (SDS-PAGE) or Western blotting with Anti-His-tag mAb-HRP-DirecT (Medical & Biological Laboratories, Nagoya, Japan).

### Substrate preparation

Extensin was extracted from carrot and Hyp-linked ß-l-arabino-oligosaccharides (ß-Ara_2_, Ara_2_-Hyp, Ara_3_-Hyp, and Ara_4_-Hyp) were prepared as described previously [2]. To simplify the assay for enzymatic activity, dansylated Hyp-linked ß-L-arabino-oligosaccharides (Ara_2_-Hyp-DNS, Ara_3_-Hyp-DNS and Ara_4_-Hyp-DNS) were prepared as described by Gray [30].

### Enzymatic assays

A 10-μl reaction mixture for thin-layer chromatography (TLC) analysis of dansylated substrates contained 50 mM sodium acetate buffer (pH 4.5), 50 μM substrate, and 1 μL of the expressed recombinant enzyme. After 12 h at 30°C, the reaction mixtures were spotted on a Silica Gel 60 aluminum plate (Merck, Darmstadt, Germany) and developed with a 3:1:1 solvent (v/v/v) of 1-butanol/acetic acid/water and finally visualized with UV light. For orcinol-stained TLC analysis, the 100-μL reaction mixture contained 50 mM sodium acetate buffer (pH 4.5), 35 μM substrate, and 2 μL of an expressed enzyme, and the reaction was conducted at 30°C for 12 h. Spotted silica gels were developed with a 2:1:1 solvent (v/v/v) of ethyl acetate/acetic acid/water. Sugars were visualized by spraying an orcinol—sulfate reagent onto the silica gel plate [31]. For high-performance anion-exchange chromatography with pulsed amperometric detection (HPAEC-PAD) analysis, oligosaccharides in the 100-μL reaction mixture were analyzed with a CarboPac PA-1 column. The column was eluted at a flow rate of 1.0 mL/min with the following gradient: 0–5 min, 100% eluent A (0.1 M NaOH); 5–30 min, 0–100% eluent B (0.5 M sodium acetate and 0.1 M NaOH); and 30–35 min, 100% eluent B.

### RNA expression analysis

Total RNA from *X*. *euvesicatoria* UPB139 was extracted using the Nucleospin RNA II (MACHERY-NAGEL, Duren, Germany) according to the manufacturer’s instructions, except that the phenol—chloroform—isoamyl alcohol mixture was used only when inoculated plants were macerated in the first step. To confirm the absence of any genomic DNA contamination, extracted RNAs were directly subjected to PCR. DNA-free RNA was then converted to cDNA using the ReverTra Ace (Toyobo, Osaka, Japan) with random hexamers. Quantitative reverse-transcription PCR (qRT-PCR) was conducted in a LightCycler Nano System (Roche Diagnostics, Rotkreuz, Switzerland) with TB Green *Premix Ex Taq* II (Takara Bio). Cycling conditions were initial denaturation for 2 min at 95°C; 45 cycles of 95°C for 15 s, 55 °C for 15 s, and 72°C for 15 s. Relative levels of gene expression were calculated using the 2^-ΔΔ*C*t^ method [32]. For each amplification run, the calculated threshold cycle for each gene amplification was normalized against that of the reference gene 16S rRNA. Three technical replicates were performed each time. Primers used in this analysis are shown in S1 Table.

### Generation of gene-deletion mutants

*X*. *euvesicatoria* mutants were constructed via double homologous recombination using the suicide vector pK18mobSacB [27], which harbors the *sacB* gene as a counterselection marker. All primers used in this experiment are shown in S1 Table. Single-gene deletion mutants of *xehypBA1*, *xehypAA*, and *xehypBA2* were generated as below. For the *xehypBA1* deletion, the two primer pairs, DIS1/DIS2 containing the *Eco*RI site and DIS3/DIS4 containing the *Xba*I site were used to amplify the 492-bp upstream region and the 590-bp internal region, respectively. The upstream region was first cloned into the *Eco*RI site of pK18mobSacB via In-Fusion HD Cloning kit (Takara Bio), while the internal region was cloned into the *Xba*I site in the same manner, yielding pK18-xcv2724. Similarly, for the *xehypAA* deletion, primer pairs DIS13/DIS14 and DIS15/DIS16 were used to amplify the 584-bp and 510-bp fragments and cloned into pK18mobSacB, yielding pK18-xcv2728. For the *xehypBA2* deletion, primer pairs, DIS7/DIS8 and DIS9/DIS10 were used to amplify the 517-bp and 591-bp fragments and cloned into pK18mobSacB, yielding pK18-xcv2729. For the *xehypAA*-*xehypBA2* double deletion, primer pairs DIS7/DIS8 and DIS15/DIS16, described above were used to construct vector pK18-xcv2728-2729. For the *xehypBA1*-*xehypAA*-*xehypBA2* triple deletion, the double deletion mutant was used as a recipient and the *xehypBA1* gene was deleted in the manner described above. The *hrpX*-deletion mutant of *X*. *euvesicatoria* was created using primer pairs DIS19/DIS20 and DIS21/DIS22 to amplify the 500-bp and 419-bp fragments, which were then cloned into pK18mobSacB, yielding pK18-hrpX.

The deletion vectors were inserted into *E*. *coli* S17-1 (*λpir*) cells and then introduced into *X*. *euvesicatoria* by biparental conjugation. Crystal violet (0.3% w/v) was used to select a kanamycin-resistant isolate *X*. *euvesicatoria*, discriminating it from a kanamycin-resistant *E*. *coli* for the first screening. A marker-exchanged mutant was obtained by double homologous recombination using sucrose selection (*SacB*) as reported previously [33]. Deletion mutants were confirmed by PCR and sequencing.

### Inoculation tests

Micro-Tom plants [34] were grown from seeds in a plant growth chamber at 28°C with 16 h light/8 h dark. Plants 4–5 weeks old were dipped into a suspension of bacteria (OD_600_ of 0.1) containing 0.02% (v/v) surfactant Silwet L-77 and 10 mM MgCl_2_. Plants were then covered with plastic bags for 48 h to maintain a moist environment, grown for 10 more days in the chamber, and checked for symptoms. Leaves of other 4–5-week-old plants were infiltrated with a 100-fold dilution of a bacterial suspension grown to an OD_600_ of 0.1.

For assessing nonhost responses, tobacco plants (*Nicotiana tabacum*) were grown in a greenhouse up to the 4–5 leaf stage and transferred to a growth chamber at 25°C under a 16-h light/8-dark 3 days before inoculation. Leaves were infiltrated with a bacterial suspension (OD_600_ of 0.4) in 10 mM MgCl_2_ using a syringe. Inoculated plants were then grown in the chamber for another 3 days and checked for symptoms.

## Results

### Expression and purification of XeHypBA1, XeHypBA2, and XeHypAA

**Fig 2:**
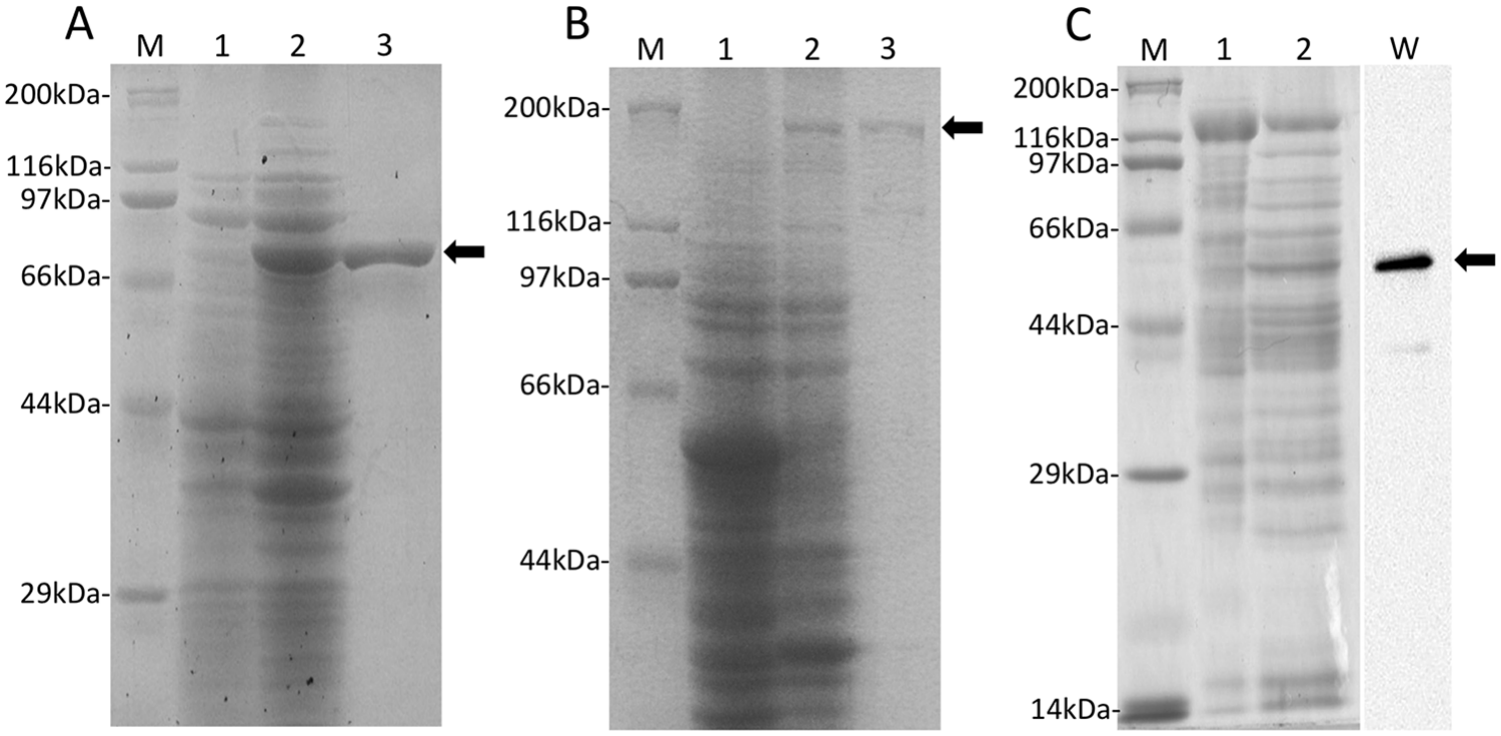
SDS-PAGE analysis of expressed enzymes. XeHypBA1 (A), XeHypBA2 (B), XeHypAA (C). M, protein molecular weight marker; lane 1, soluble proteins from mock vectors; lane 2, soluble proteins from expression vectors; lane 3, His-tagged proteins; W, western blots for the purified protein. *Arrows* indicate purified proteins.

XeHypBA1 consisted of 791 aa. The recombinant protein without the signal peptide (43 aa) was expressed in *E*. *coli* BL21 (λDE3) and obtained as a soluble protein. SDS-PAGE showed that the purified recombinant XeHypBA1 protein migrated as a single band with an estimated molecular mass of 82.8 kDa (Fig 2A).

XeHypBA2 consisted of 1452 aa. The recombinant protein without the signal peptide (42 aa) was expressed in *E*. *coli* BL21 (λDE3) as a fusion protein, in which the protein of interest was fused with the trigger factor chaperone because a soluble protein was not obtained without a trigger that induces protein solubility. SDS-PAGE showed one band with a slightly lower molecular mass than the expected size 200 kDa (XeHypBA2, 152 kDa; and trigger factor, 48 kDa; Fig 2B).

XeHypAA consisted of 528 aa, and the recombinant protein without the signal peptide (24 aa) was expressed in *B*. *choshinensis* SP3 as a secretory protein because we could not obtain a soluble protein in *E*. *coli* even with the trigger factor. The expression level of XeHypAA in *B*. *choshinensis* was low, and the purified protein was not visible in an SDS-PAGE gel stained with Coomassie brilliant blue. Thus, we used Western blotting with His-tag antibodies to confirm expression of the target protein (Fig 2C). Western blotting showed a His-tagged protein with an estimated molecular mass of 55.3 kDa (Fig 2C).

### Substrate specificity of XeHypBA1, XeHypBA2, and XeHypAA

**Fig 3:**
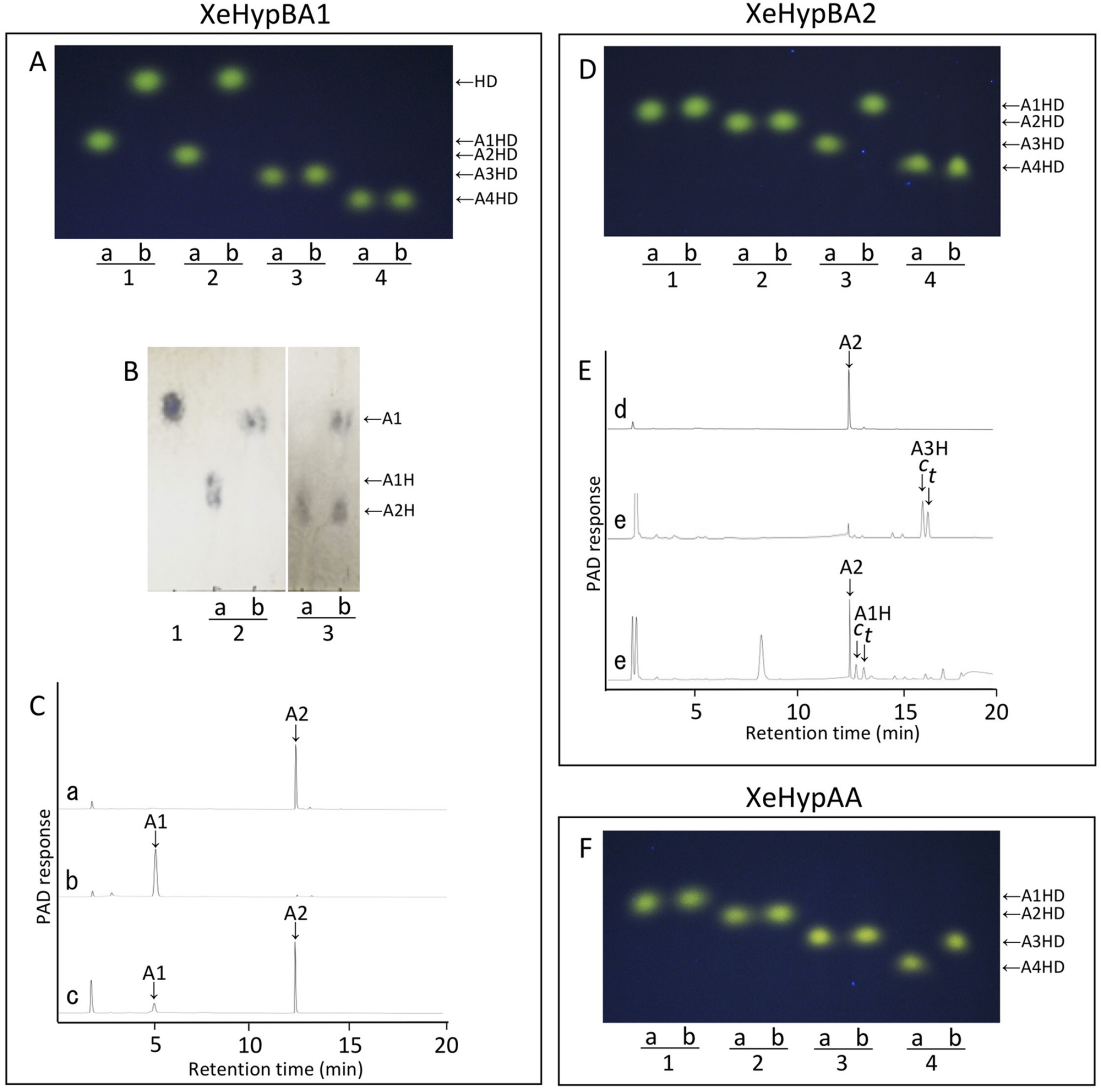
Substrate specificity of XeHypBA1 (A, B, C), XeHypBA2 (D, E) and XeHypAA (F). (A, D, F) Dansylated (DNS) *cis*-substrates were incubated either without (lane a) or with (lane b) XeHypBA1, XeHypBA2, or XeHypAA and the reaction products were analyzed by TLC. Ara-Hyp-DNS (lane 1), Ara_2_-Hyp-DNS (lane 2), Ara_3_-Hyp-DNS (lane 3), and Ara_4_-Hyp-DNS (lane 4) were used as substrates. (B) TLC analysis of XeHypBA1 reaction products. l -arabinose standard (lane 1). Ara-Hyp (lane 2) and Ara_2_-Hyp (lane 3) were incubated either without (lane a) or with (lane b) XeHypBA1. (C, E) HPAEC-PAD analysis of XeHypBA1or XeHypBA2 reaction products. β-Ara_2_ standard (a, d); l-arabinose standard (b); Ara_3_-Hyp standard (e); β-Ara_2_ incubated with XeHypBA1 (c); Ara_3_-Hyp incubated with XeHypBA2 (e). HD, Hyp-DNS; A1HD, Ara-Hyp-DNS; A2HD, Ara_2_-Hyp-DNS; A3HD, Ara_3_-Hyp-DNS; A4HD, Ara_4_-Hyp-DNS. A1, L-arabinose; A1H, Ara-Hyp; A2, β-Ara_2_; A2H, Ara_2_-Hyp; A3H, Ara_3_-Hyp; *c*, *cis*-isomer; *t*, *trans*-isomer.

XeHypBA1 preferred Ara-Hyp as a substrate and liberated L-arabinose (Fig 3A and 3B) and was only slightly active on ß-Ara_2_ (Fig 3C), but did not use Ara_4_-Hyp and Ara_3_-Hyp at all. Ara_2_-Hyp was completely degraded in its dansylated form, but only partially when unmodified (Fig 3A and 3B). These activities differ from those of HypBA1 from *B*. *longum*, which degrade ß-Ara_2_, Ara_3_-Hyp, Ara_2_-Hyp, and Ara-Hyp. These results indicate that XeHypBA1 mainly acts on Ara-Hyp.

XeHypBA2 catalyzed the hydrolysis of Ara_3_-Hyp (Fig 3D), liberating ß-Ara_2_ (arabinobiose) (Fig 3E). However, Ara_4_-Hyp, Ara_3_-Hyp, and Ara-Hyp were not hydrolyzed. XeHypBA2 is thus a ß-l-arabinobiosidase that has strict substrate specificity for Ara_3_-Hyp. These activities were also the same as HypBA2 from *B*. *longum*.

XeHypAA specifically degraded Ara_4_-Hyp to Ara_3_-Hyp and L-arabinose (Fig 3F). The enzyme did not hydrolyze Ara_3_-Hyp, Ara_2_-Hyp, and Ara-Hyp, indicating that XeHypAA is an α1,3-specific α-l-arabinofuranosidase, which recognizes the Ara*f*-α1,3-Ara*f* structure of Ara_4_-Hyp. These activities were exactly the same as found for HypAA from *B*. *longum*.

**Fig 4:**
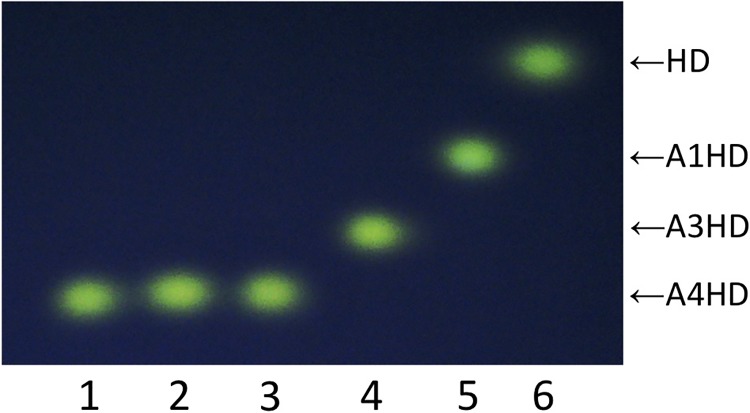
TLC analysis of reactions by activity from various combinations of XeHypBA1, XeHypBA2, and XeHypAA. Ara_4_-Hyp-DNS was incubated either without any enzymes (lane 1), or with XeHypBA1 (lane 2), with XeHypBA2 (lane 3), with XeHypAA (lane 4), with XeHypBA2 and XeHypAA (lane 5), or with XeHypBA1, XeHypBA2, and XeHypAA (lane 6). HD, Hyp-DNS; A1HD, Ara-Hyp-DNS; A3HD, Ara_3_-Hyp-DNS; A4HD, Ara_4_-Hyp-DNS.

We also examined the synergistic effects of the three enzymes on the degradation of Hyp-linked arabino-oligosaccharides. Ara_4_-Hyp was completely degraded by combined activity of XeHypAA, XeHypBA2, and XeHypBA1 (Fig 4).

**Fig 5:**
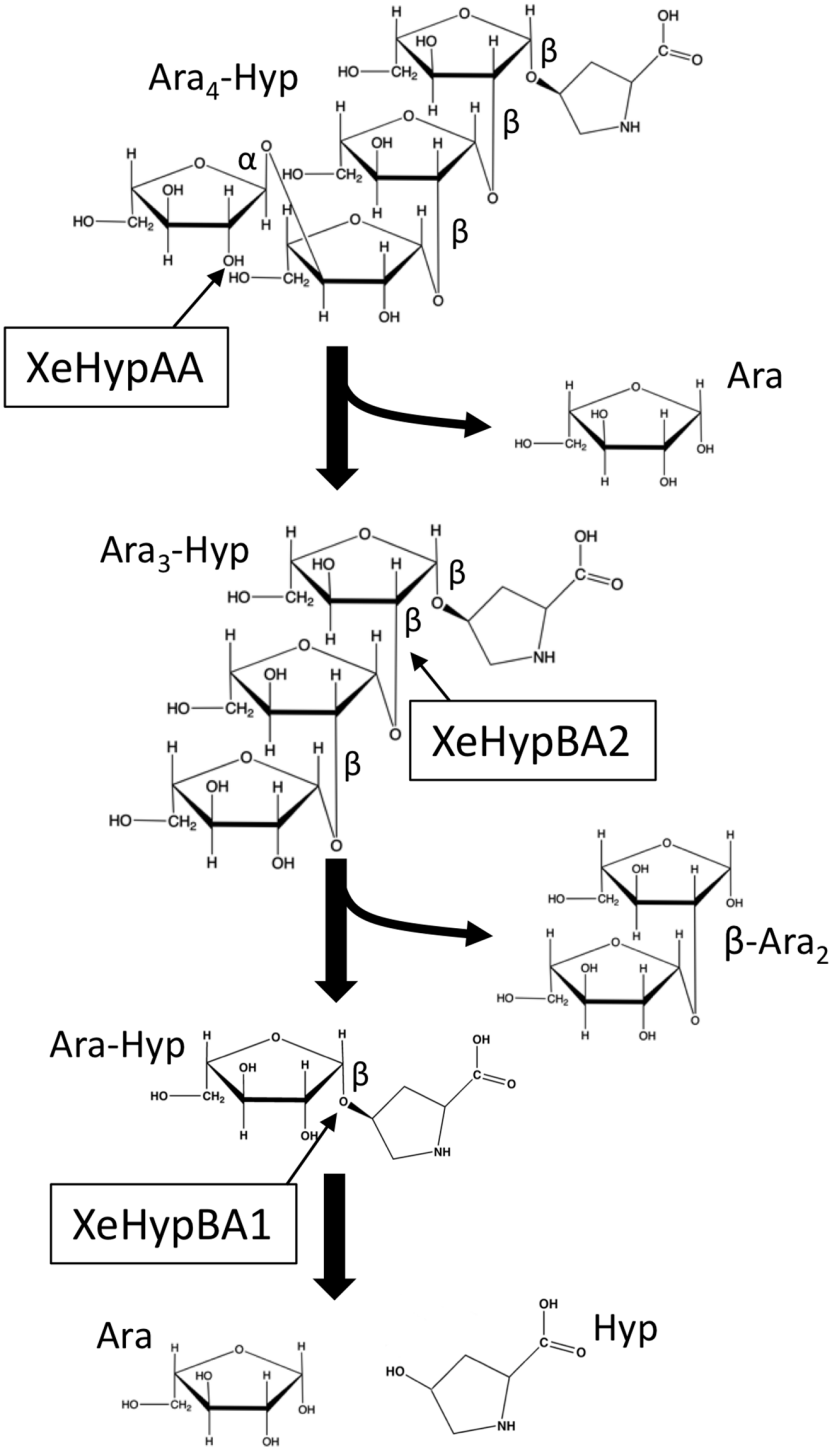
Schematic flow chart of hydrolysis of Ara_4_-Hyp by XeHypAA, XeHypBA2, and XeHypBA1. *Thin arrows* indicate cleavage sites for the enzymes.

The substrate specificities of each enzyme are summarized in Table 2, and the coordinated degradation of the oligosaccharides is shown in Fig 5.

**Table 2:** Substrate specificity of the enzymes.

		Substrates				
Enzymes	Classification	Ara_4_-Hyp	Ara_3_-Hyp	Ara_2_-Hyp	Ara-Hyp	ß-Ara_2_
XeHypAA	α-l-arabinofuronasidase	+	-	-	-	-
XeHypBA2	ß-l-arabinobiosidase	-	+	-	-	-
XeHypBA1	ß-l-arabinofuronasidase	-	-	+/-	+	+/-

+, well hydrolyzed; +/-, weakly hydrolyzed; -, not hydrolyzed

### Expression analysis of *xehypBA2-AA* operon

**Fig 6:**
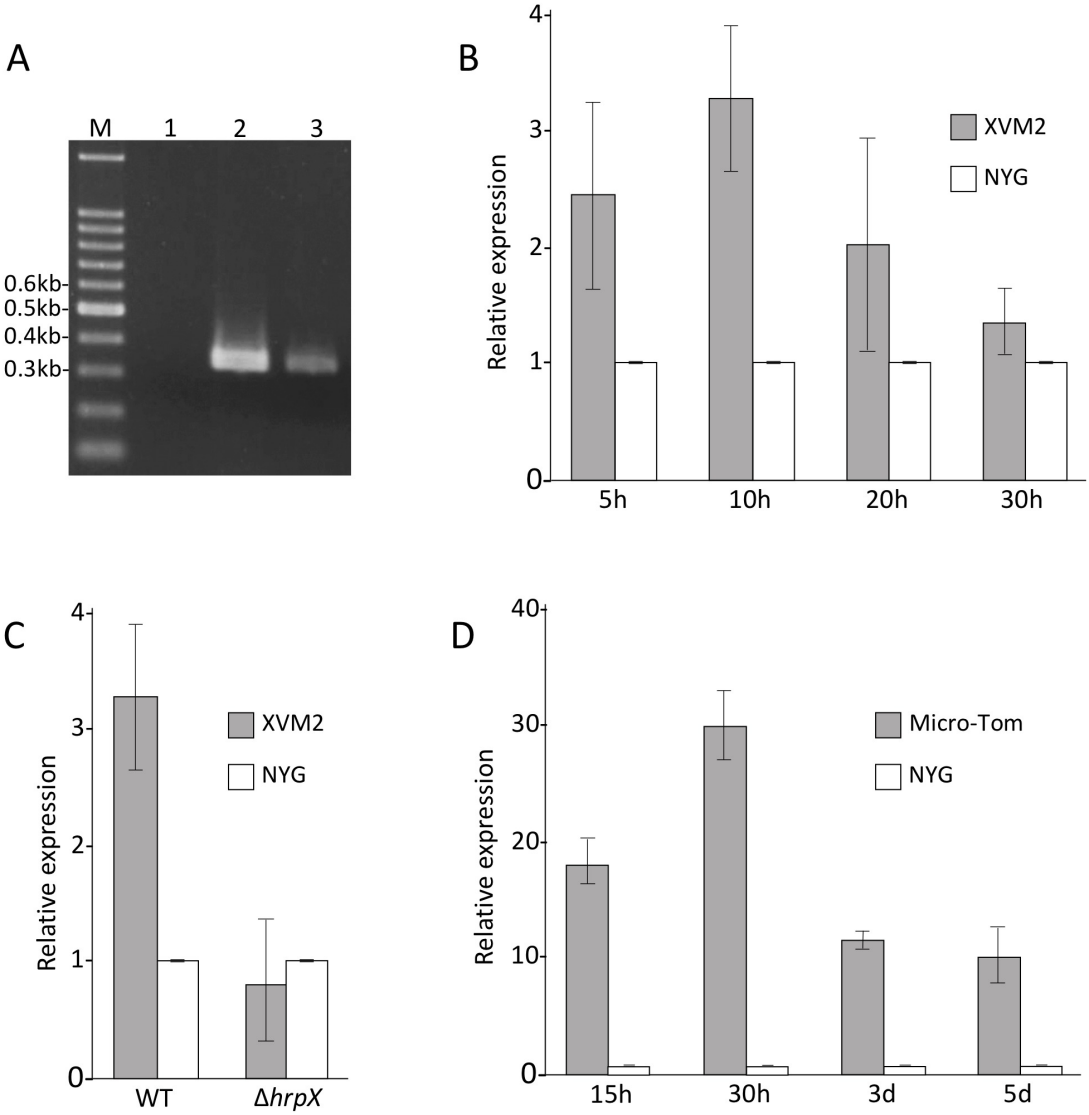
Expression analysis of *xehypBA2-AA* operon. (A) RT-PCR was conducted to confirm whether the two genes, *xehypBA2 and xehypAA*, form an operon. A 346-bp fragment containing the junction region of the two genes was amplified using extracted mRNAs (lane 1), genomic DNAs (lane 2) and cDNAs (lane 3) derived from *X*. *euvesicatoria* UPB139 grown in XVM2 (*hrp*-inducing medium). M, DNA molecular weight marker. (B) Relative expression level of the operon in NYG (complete medium) and XVM2 (*hrp*-inducing medium) was examined by qRT-PCR. (C) Relative expression level of the operon in the wild type (WT) and a *hrpX*-deletion mutant (Δ*hrpX*) in NYG and XVM2. (D) Relative expression level of the operon in NYG and infected Micro-Tom. The expression values relative to the mean expression in NYG were calculated using the 2^-ΔΔ*C*t^ method. *Error bars* indicate standard deviation (±SD) of three independent experiments.

The stop codon of *xehypBA2* and the start codon of *xehypAA* are overlapped, typical of an operon gene. Thus, to confirm whether the two genes form an operon, we conducted RT-PCR using primers EXG1 and EXG2 (S1 Table), designed to amplify a 346-bp fragment containing the junction region of the two genes. Expected fragments were obtained from cDNAs derived from the bacteria in XVM2 (*hrp*-inducing medium) (Fig 6A), indicating that *xehypBA2* and *xehypAA* are transcribed into a single mRNA. No PCR product was obtained from extracted RNAs, demonstrating the absence of genomic DNA contamination in the RNAs (Fig 6A).

In the promoter region of the *xehypBA2-AA* operon, a conserved *cis*-regulatory element, PIP box with the consensus sequence TTCGCN_15_-TTCGC [17, 35] was found. Thus, to investigate whether the operon was regulated by HrpX, we analyzed the expression of the operon gene over time using qRT-PCR; expression in XVM2 reached a maximum at 10 h and was significantly higher than in NYG (complete medium) (Fig 6B). Next, we compared the expression level of the wild type with that of the *hrpX*-deletion mutant in XVM2 after a 10-h incubation. The Δ*hrpX* mutant was generated as shown in S1 Fig. The expression level of the gene in the wild type was significantly higher than in the Δ*hrpX* mutant (Fig 6C), indicating that the gene was upregulated by HrpX. We also analyzed the expression of the gene in infected plants (Micro-Tom). The expression was much higher than in NYG at all times tested (15 h–5 days), and was highest at 30 h after inoculation (Fig 6D). Obviously, the expression of the operon gene was induced in infected plants.

### Pathogenicity of mutants

**Fig 7:**
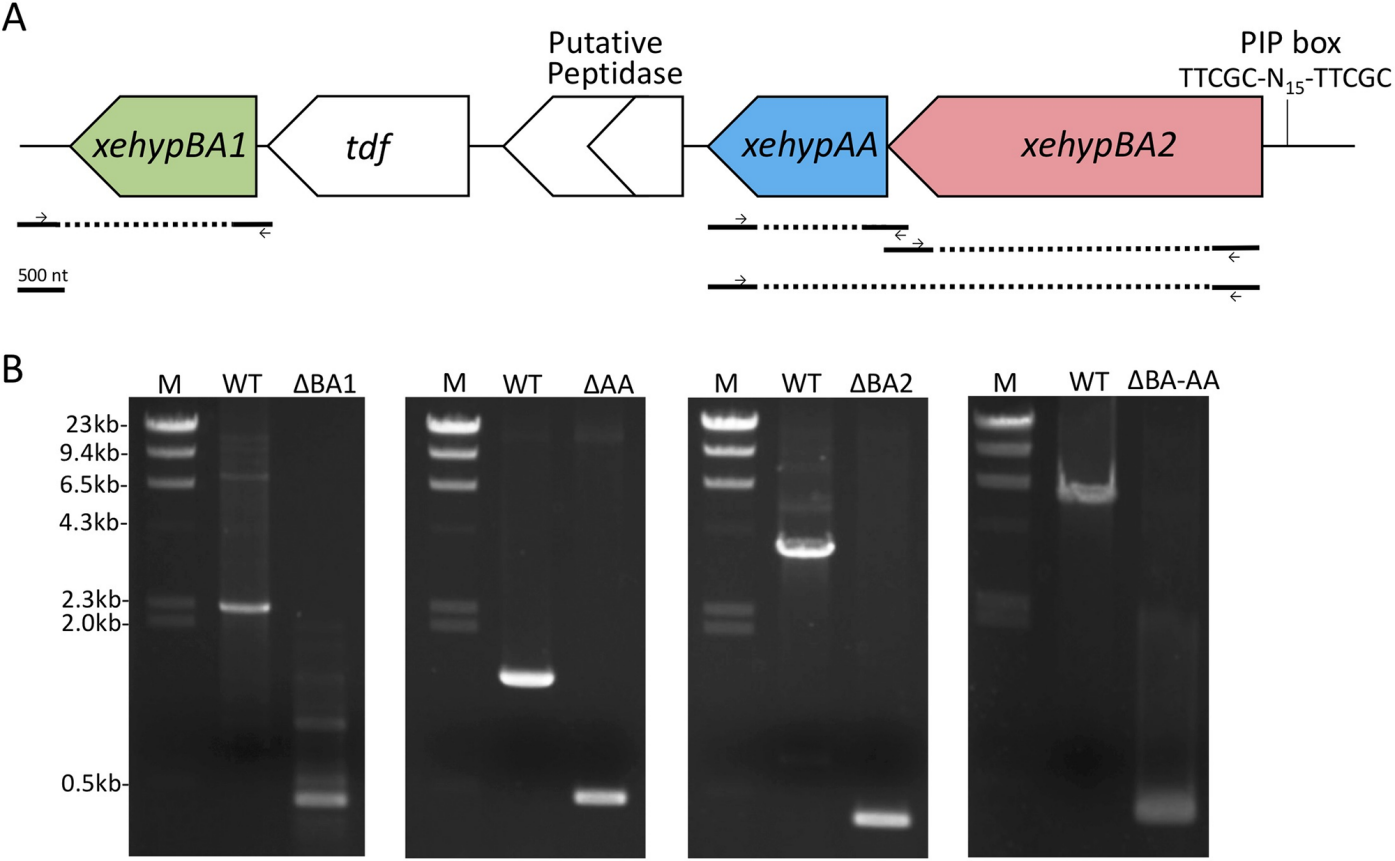
Isolation of gene-deletion mutants. (A) Genetic map of *xehypBA1*, *xehypBA2*, and *xehypAA* of *X*. *euvesicatoria*. *Solid lines* below the map represent fragments used to construct mutants. *Dashed lines* indicate the deletion regions. *Arrows* indicate primers used to confirm the gene deletion. PIP box, plant-inducible promoter box. (B) Confirmation of gene-deletion mutants by PCR. M, DNA molecular weight marker; WT, wild type; ΔBA1, Δ*xehypBA1*; ΔBA2, Δ*xehypBA2*; ΔBA-AA, Δ*xehypBA1-BA2-AA* (constructed from Δ*xehypBA1*).

Because the *xehypBA2-AA* operon was regulated by HrpX, we constructed a gene-deletion mutant of the operon gene, as well as that of *xehypBA1* to investigate whether these genes are involved in pathogenicity. Single-gene deletion mutants of each gene (Δ*xehypBA1*, Δ*xehypBA2*, Δ*xehypAA*) and a triple-gene deletion mutant of the three genes (Δ*xehypBA1-BA2-AA*) were created through biparental mating. The triple-gene deletion mutant was constructed from the Δ*xehypBA1* mutant. The obtained mutants were confirmed by the reduced size of the PCR products (Fig 7).

**Fig 8:**
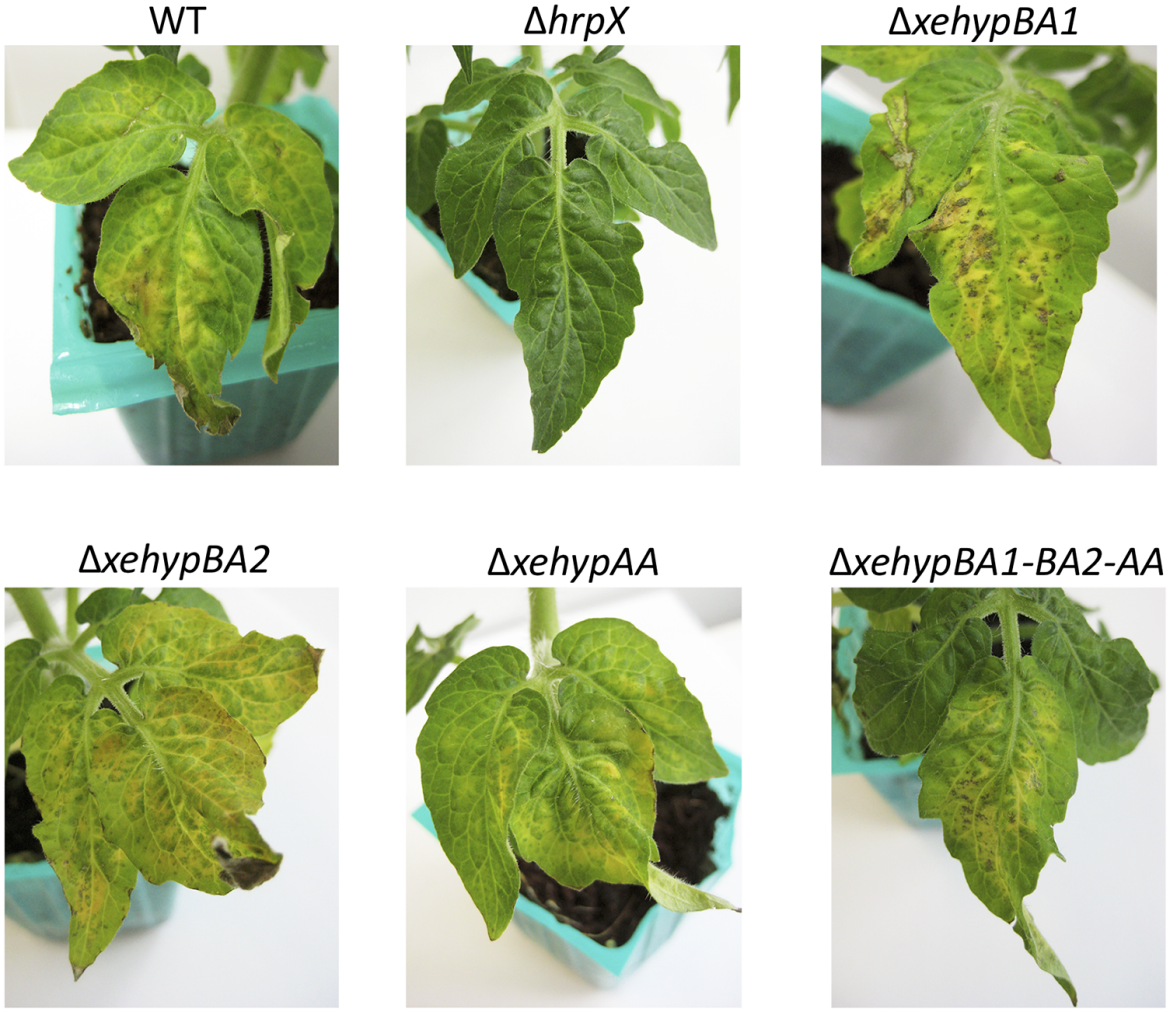
Pathogenicity test of gene-deletion mutants. Micro-Tom plants were dip-inoculated with the wild type (WT) *X*. *euvesicatoria* or the respective mutants and examined for symptoms after 12 days.

Micro-Tom plants were inoculated with one of the various mutants using the dipping method (see Materials and methods), and plants were examined for symptoms 12 days later. Single-gene deletion mutants, and even the triple-gene deletion mutant, induced the same symptoms on leaves as on the wild type (Fig 8). The double-gene deletion mutant (Δ*xehypBA2*-*AA*) also maintained pathogenicity (data not shown). The *hrpX*-deletion mutant (Δ*hrpX*) did not cause any symptoms (Fig 8). These results indicate that the three genes are not involved in the pathogenicity of *X*. *euvesicatoria*.

**Fig 9:**
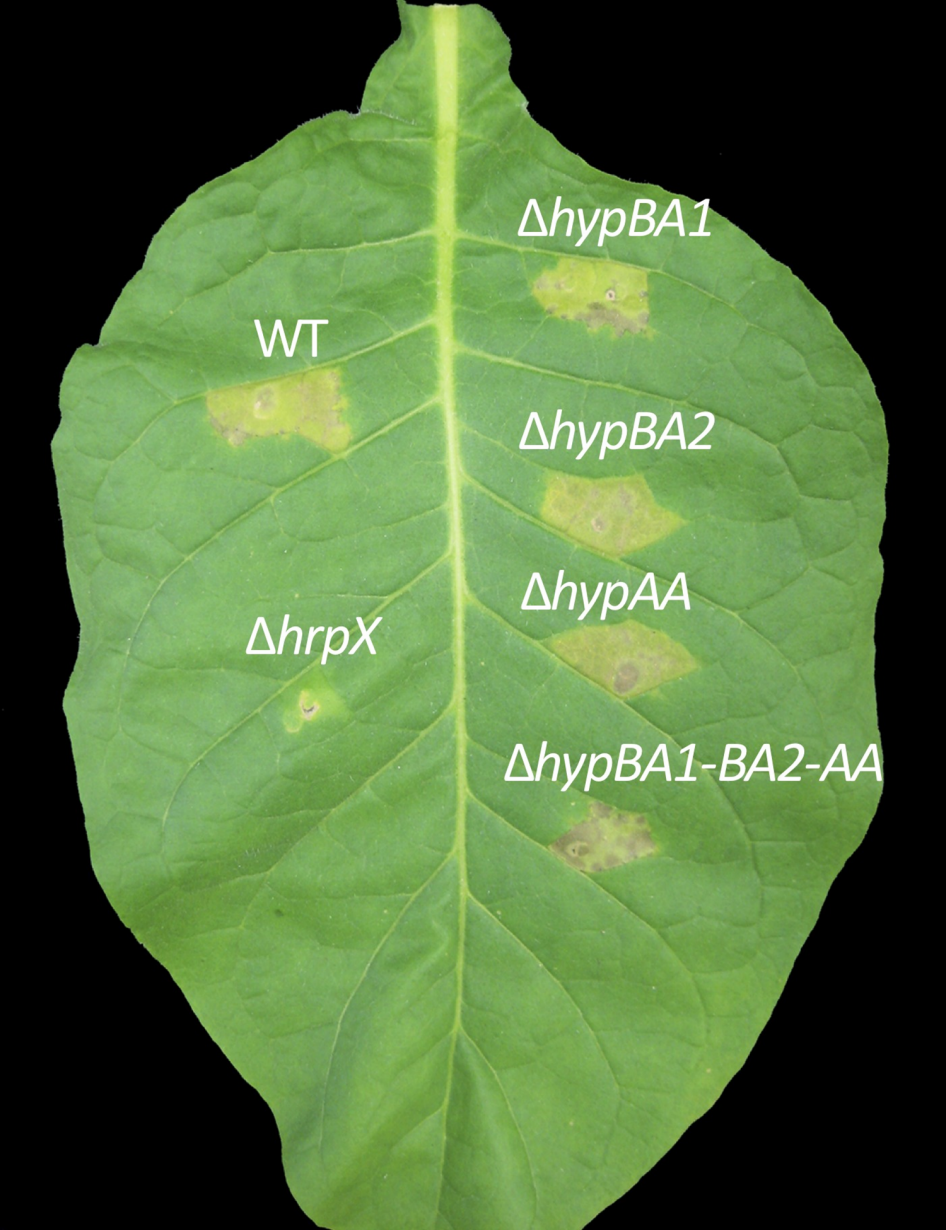
Nonhost responses in tobacco (*N*. *tabacum*) 96 h after inoculation with gene-deletion mutants. Tobacco plants at the 4–5-leaf stage were infiltrated via a syringe with the wild-type *X*. *euvesicatoria* (WT) or the respective mutants at an OD_600_ of 0.4 in 10 mM MgCl_2_.

The influence of the respective gene-deletion mutants on nonhost responses was tested by inoculating *N*. *tabacum* plants. All the mutants except for Δ*hrpX* induced chlorotic reactions in the same manner as the wild type (Fig 9), indicating that the three genes have no effect on nonhost resistance.

## Discussion

Because the genes in *B*. *longum* that encode the enzymes to degrade arabino-oligosaccharides on HRGPs are found only in *Xanthomonas* spp. among plant pathogenic bacteria [1, 2], we cloned and characterized the homologous genes *xehypBA1*, *xehypBA2*, and *xehypAA* derived from *X*. *euvesicatoria* that infects tomato and pepper to better understand the role of these enzymes in pathogenicity of the bacterium.

The gene *xehypBA1* is the homolog of *hypBA1* from *B*. *longum*. HypBA1, assigned to the new GH family 127, is a novel ß-l-arabinofuranosidase that liberates L-arabinose from ß-Ara_2_, Ara-Hyp, Ara_2_-Hyp, and Ara_3_-Hyp [1]. However, XeHypBA1 liberated L-arabinose specifically from Ara-Hyp and slightly from ß-Ara_2_ and Ara_2_-Hyp; the enzyme likely works better on Ara_2_-Hyp-DNS than Ara_2_-Hyp (unmodified form) because the conformation of Ara_2_-Hyp-DNS may provide a better fit with the enzyme than that of Ara_2_-Hyp (Fig 3A, 3B and 3C), but the cause is unclear. HypBA1 from *B*. *longum* is an intracellular protein; *B*. *longum* takes up ß-Ara_2_ and degrades the disaccharide in the cells. XeHpyBA1, on the other hand, is an extracellular protein and can work directly on the arabino-oligosaccharides on HRGPs (data not shown). These results indicate that XeHpyBA1 is quite different from HypBA1.

The gene *xehypBA2* is the homolog of *hypBA2* from *B*. *longum*. Because the recombinant protein of XeHypBA2 was not expressed in the pET system as a soluble protein, we used the pCold TF system that expresses a fusion protein with the trigger factor chaperon as a soluble protein. In the SDS-PAGE analysis, the fusion protein migrated at a slightly lower molecular mass than expected (Fig 2B), probably because the fusion protein was not completely unfolded by the SDS even after extensive heating due to the complexity of the protein structure. HypBA2 from *B*. *longum*, assigned to the new HG family 121, is a novel ß-l-arabinobiosidase that has strict substrate specificity for Ara_3_-Hyp [2]. It is an extracellular protein with a membrane-anchoring region (MAR) at its C-terminal region, suggesting that the enzyme may be on the bacterial surface and that it liberates ß-Ara_2_ from HRGPs adjacent to the cells. XeHypBA2 also liberates ß-Ara_2_ only from Ara_3_-Hyp, not from Ara_2_-Hyp and Ara_4_-Hyp, so it also is highly specific for the structure of Ara_3_-Hyp (Fig 3D and 3E). It is also an extracellular protein, but it has no MAR, indicating that the secreted enzyme freely degrades Ara_3_-Hyp on HRGPs.

The gene *xehypAA* is the homolog of *hypAA* from *B*. *longum*. The recombinant protein of XeHypAA was not expressed either in the pET system or even in the pCold TF system. We then used the *Brevibacillus* expression system, which is well-suited for heterologous protein expression. HypAA from *B*. *longum*, containing a GH43 domain, is an α1,3-specific α-l-arabinofuranosidase, which recognizes the Ara*f*-α1,3-Ara*f* structure of Ara_4_-Hyp [unpublished data]. XeHypAA also specifically degraded Ara_4_-Hyp to Ara_3_-Hyp and L-arabinose, recognizing the α1,3 bond of arabinose that is only on Ara_4_-Hyp (Fig 3F). HypAA is a secretory enzyme with MAR at its C-terminal region like HypBA2, but XeHypAA has no MAR. XeHypAA also may freely degrade Ara_4_-Hyp on HRGPs.

When we examined the synergistic effects among various combinations of the three enzymes on the degradation of Ara_4_-Hyp (Fig 4), they coordinately degraded Ara_4_-Hyp (Fig 5). Ara_4_-Hyp and Ara_3_-Hyp are the major Hyp-linked l-arabinofuranosides in dicotyledons. In particular, Ara_4_-Hyp accounts for 33–75% of the total Hyp residues in plant cell walls [16]. Because XeHypBA2 cannot directly hydrolyze Ara_4_-Hyp, XeHypAA is required for further degradation. Thus, the operon construction of the two genes, *xehypBA2* and *xehypAA*, is quite reasonable.

With regard to the involvement of HRGPs of extensin and lectins in plant defense [3–9], Brown *et al*. [36] reported that, in the pepper–*X*. *campestris* interaction, restriction of bacterial colony development was linked to the formation of an amorphous papillae-like matrix containing HRGPs around bacterial cells. Because the enzymes from *X*. *euvesicatoria* can remove the arabinofurano-oligosaccharides on HRGPs, we expected that deglycosylated HRGPs would be unstable and degradable by proteases, leading to a reduction in plant resistance. As a matter of fact, in the promoter region of the operon gene (*xehypBA2*-*AA*), there is a PIP box, which binds with the global-regulator HrpX to regulate pathogenicity-related genes. In the qRT-PCR for the operon gene using a *hrpX*-deletion mutant, the gene was evidently regulated by HrpX (Fig 6C). Koebnik *et al*. [17] also reported that expression of *xcv2729* (*xehypBA2*) was dependent on HrpG and HrpX. Furthermore, the gene was upregulated in inoculated plants (Fig 6D). In the qRT-PCR of *xehypBA1*, surprisingly, the gene was also upregulated in infected plants, even though there were no PIP box-like sequences in the promoter region (data not shown). These data strongly suggest that the genes are involved in pathogenicity. Thus, we created in-frame deletion mutants for each enzyme gene. However, in contrast to expectations, the single-gene deletion mutants and even the triple-gene mutant remained pathogenic (Fig 8). We also investigated whether the mutants exert an influence on the resistance reactions of a nonhost plant because of the possibility that the monosaccharides (Ara) or disaccharides (ß-Ara_2_) freed by the enzymes might act as an elicitor. However, the inoculated tobacco plants (*N*. *tabacum*) developed chlorosis (not fast cell death) in the areas surrounded by veins (Fig 9), typical resistance reactions of *N*. *tabacum* against *X*. *euvesicatoria* [37]. These results indicate that the enzymes are not involved in either pathogenicity or nonhost resistance reactions.

*B*. *longum* uses these enzymes to degrade the arabinofurano-oligosaccharides on HRGPs that reach the intestine and thus frees L-arabinose for use as their carbohydrate source [1]. *X*. *euvesicatoria* cannot utilize L-arabinose as a carbon source [38], and as described above, the enzymes are not involved in pathogenicity. So why does *X*. *euvesicatoria* have these enzymes? Besides their presence in *X*. *euvesicatoria*, homologs of *hypBA2* and *hypAA* are conserved in only two species of *Bifidobacterium* (*B*. *longum* and *B*. *pseudocatenulatum*), but not in other intestinal bacteria such as *Bacteroides*, *Salmonella*, *Clostridium*, and *Escherichia* [2]. The homologs are also found in some actinomycetes such as *Streptosporangium roseum*, *Actinosynnema mirum*, and *Micromonospora aurantiaca* [2]. On the other hand, in *Xanthomonas* spp., homologs are conserved in 12 of 13 species deposited in public databases but not in other plant pathogenic bacteria such as *Pseudomonas*, *Erwinia*, *Pectobacterium*, and *Burkholderia*. In sum, the genes are distributed among some species in several genera of bacteria, but are well conserved in the species of *Xanthomonas*. Therefore, these genes might have been transferred horizontally from *Xanthomonas* to bifidobacteria or actinomycetes. If *Xanthomonas* spp. had acquired these genes originally, what role did they originally play? Initially, these enzymes may have contributed to pathogenicity, but plants may have overcome it, and in response the bacteria evolved other pathogenicity factors to cause disease. In other words, these enzymes may now be useless for *Xanthomonas* spp. We also deleted the homologous genes from *X*. *campestris* pv. *campestris* that infects cruciferous plants, and the deletion did not alter its pathogenicity (data not shown), just as we found for *X*. *euvesicatoria*. Recently, the full genome sequence of *X*. *phaseoli* that infects common bean, *Phaseolus vulgaris* has become available, and the homolog of *xehypBA2* in *X*. *phaseoli* was annotated as a pseudogene (XppCFBP6546P_19765) [39]. A putative peptidase gene located adjacent to *xehypAA* from *X*. *euvesicatoria* is predicted to encode a prolyl oligopeptidase to hydrolyze proline-containing peptides (Fig 7A). The enzyme may be able to degrade HRGPs rich in hydroxyproline. However, a frameshift mutation has been caused by a single nucleotide deletion, creating a stop codon in the internal region, indicating that the gene has also become a pseudogene. These facts indicate that the genes encoding enzymes related to degradation of arabino-oligosaccharides on HRGPs may be in the process of disappearing in *Xanthomonas* spp.

## Supporting information

S1 Fig Construction of Δ*hrpX* mutant. (A) Genetic map of *hrpX* of *Xanthomonas euvesicatoria*. *Solid lines* below the map represent fragments used to construct mutants. *Dashed line* indicates the deletion region. *Arrows* indicate primers used to confirm the gene deletion. (B) Confirmation of gene deletion by PCR. M, DNA molecular weight marker; WT, wild type. (C) Confirmation of pathogenicity loss of Δ*hrpX* mutant 10 d after infiltration of Micro-Tom leaves with wild type (WT) or Δ*hrpX* mutant (no symptoms).
(TIF)

S1 Table Primers used in this study. (DOCX)
