# Supplementary figures and images for: Functional characterization of unique enzymes in Xanthomonas euvesicatoria related to degradation of arabinofurano-oligosaccharides on hydroxyproline-rich glycoproteins
Source: PLoS One. 2018 Aug 9;13(8):e0201982. doi: 10.1371/journal.pone.0201982 (PMC6085000; doi:10.1371/journal.pone.0201982)

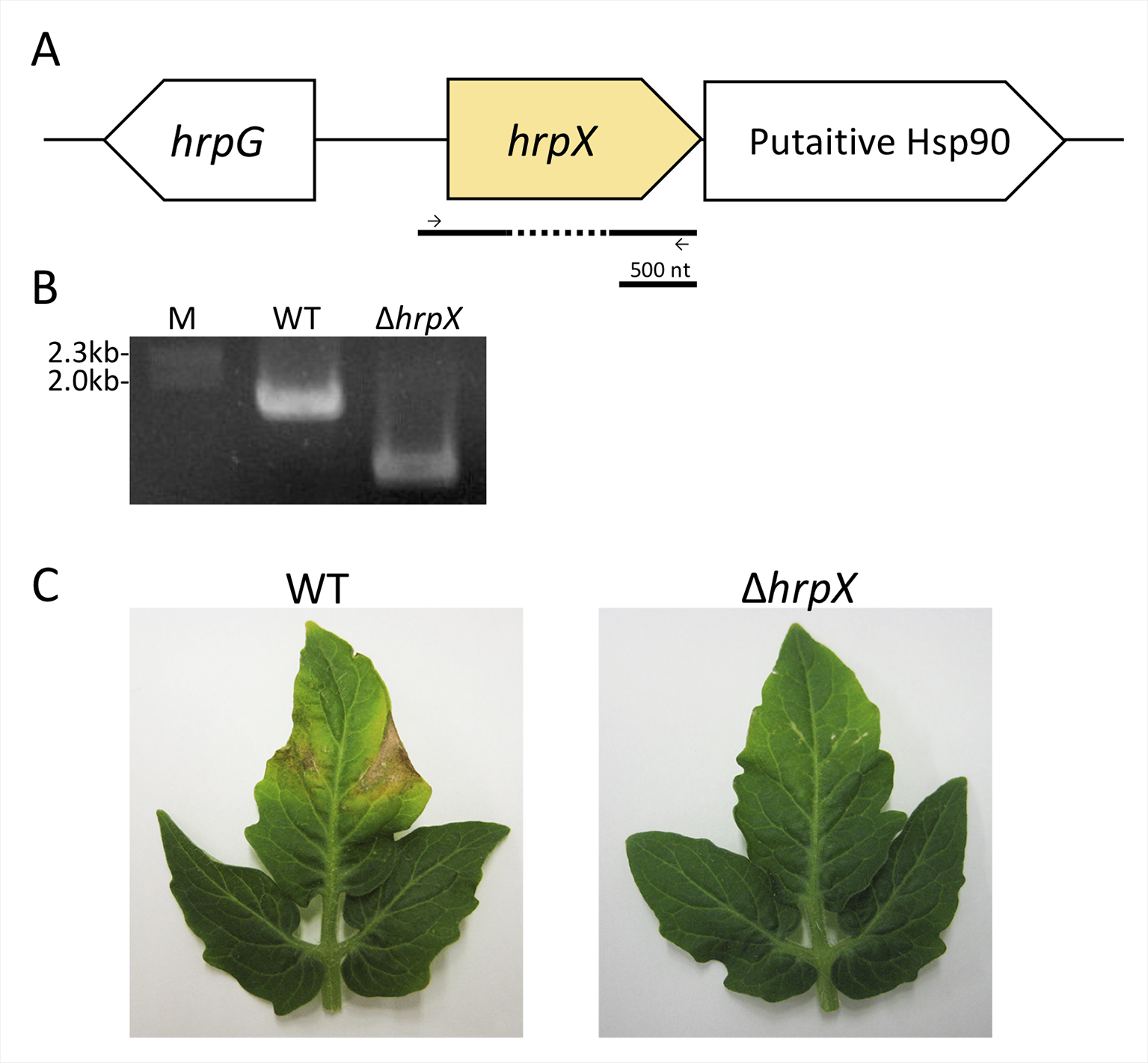

Supplement: S1 Fig — (A) Genetic map of hrpX of Xanthomonas euvesicatoria. Solid lines below the map represent fragments used to construct mutants. Dashed line indicates the deletion region. Arrows indicate primers used to confirm the gene deletion. (B) Confirmation of gene deletion by PCR. M, DNA molecular weight marker; WT, wild type. (C) Confirmation of pathogenicity loss of ΔhrpX mutant 10 d after infiltration of Micro-Tom leaves with wild type (WT) or ΔhrpX mutant (no symptoms). (TIF) [file pone.0201982.s001.tif]
