# Supplementary material for: Functional characterization of unique enzymes in Xanthomonas euvesicatoria related to degradation of arabinofurano-oligosaccharides on hydroxyproline-rich glycoproteins
Source: PLoS One. 2018 Aug 9;13(8):e0201982. doi: 10.1371/journal.pone.0201982 (PMC6085000; doi:10.1371/journal.pone.0201982)
Supplement: S1 Table — (DOCX) [file pone.0201982.s002.docx]

S1 Table. Primers used in this study

| Protein expression | | |
| --- | --- | --- |
| Primer name | Gene | Sequence (5′- 3′) |
| EXP1 | *xehypBA1* | aaggagatata *CATATG* GCACAACCGGGCAGCGTGC |
| EXP2 | *xehypBA1* | ggtggtggtg *TCGAG* CGCCGACGCCAGCACCCAA |
| EXP3 | *xehypBA2* | tcgaaggtagg *CATATG* CAGGAAGCGCCGATCGCCC |
| EXP4 | *xehypBA2* | aattcggatcc *CTCGAG* TGGCGTGGCCGCCTTGATC |
| EXP5 | *xehypAA* | gatgacgatgacaaa CAGCCGCCGCAGGGAACGCG |
| EXP6 | *xehypAA* | catcctgttaagctt TCACGGCGCATCGCAACGCT |
| Analysis of gene expression | | |
| EXG1 | *xehypBA2-AA* | TCGGGCATGCACCGACGTTA |
| EXG2 | *xehypBA2-AA* | AATATTCGCTGCCATCGGCG |
| EXG3 | *xehypBA2-AA* | ATTGGCACTCCACCGATTAC |
| EXG4 | *xehypBA2-AA* | GTCGTGAAATAACTCGGCGT |
| EXG5 | 16S rRNA | ATACCCGATTGTTCTGACGG |
| EXG6 | 16S rRNA | TTACGCCCAGTAATTCCGAG |
| EXG7 | α-tubulin | GCCTACCATGAGCAGCTTTC |
| EXG8 | α-tubulin | GAACATGAGACAGCAAGCCA |
| Construction of gene-disruption mutants | | |
| DIS1 | *xehypBA1* | accatgattac *GAATTC* CTGGGCACTGGACGACTGGA |
| DIS2 | *xehypBA1* | gtaccgagctc *GAATTC* CTCGGGTGCGCTTCTGTCAT |
| DIS3 | *xehypBA1* | cccggggatcc *TCTAGA* CAGGACATCCTGCAACGCCT |
| DIS4 | *xehypBA1* | tgcaggtcgac *TCTAGA* ACCCAACACCCAAATGCAGG |
| DIS5 | *xehypBA1* | TGGACAACATCGCCAACAAG |
| DIS6 | *xehypBA1* | AGTTCGGTCTGGCGTTGCTG |
| DIS7 | *xehypBA2* | accatgattac *GAATTC* CACGGTTCTACAGCTACTCC |
| DIS8 | *xehypBA2* | gtaccgagctc *GAATTC* TTCCAGCGTAACTCCCTCAT |
| DIS9 | *xehypBA2* | cccggggatcc *TCTAGA* ACAGGCATGGCAGCTGGATA |
| DIS10 | *xehypBA2* | tgcaggtcgac *TCTAGA* CTCTTGCTGACGATGTCCTG |
| DIS11 | *xehypBA2* | CGCAGCTATCTATTGTCCGA |
| DIS12 | *xehypBA2* | CAATGGAATGCTCTGCAGCG |
| DIS13 | *xehypAA* | accatgattac *GAATTC* GGTGTTGACGACCCAGACCA |
| DIS14 | *xehypAA* | gtaccgagctc *GAATTC* CCAGCGACCGTGAGAACGTA |
| DIS15 | *xehypAA* | cccggggatcc *TCTAGA* GGTATCTGGTGTATCACACC |
| DIS16 | *xehypAA* | tgcaggtcgac *TCTAGA* ACACCGCTTCTTCGTGATCG |
| DIS17 | *xehypAA* | TCACGGTGCAATACAACGAC |
| DIS18 | *xehypAA* | TGCTGCGGTGGATTGTTGTC |
| DIS19 | *hrpX* | accatgattac *GAATTC* ATGATCCTTTCCACCTACTTTG |
| DIS20 | *hrpX* | gtaccgagctc *GAATTC* GGAAGTGGGTGAGCGATTT |
| DIS21 | *hrpX* | cccggggatcc *TCTAGA* AGCGTTGCTGCTCTACAACC |
| DIS22 | *hrpX* | tgcaggtcgac *TCTAGA* CATTGAAGTGCTGGCGATAG |
| DIS23 | *hrpX* | ATCCGCTGCATACAATCG |
| DIS24 | *hrpX* | ACATTGCACGCACGTCTC |

Lowercase sequences are homologous to vector ends. Italicized sequences represent restriction enzyme sites.
